# Absence of steatosis combined with cardiometabolic risk factors confers the highest hepatocellular carcinoma risk in treated chronic hepatitis B

**DOI:** 10.1080/07853890.2026.2658921

**Published:** 2026-04-20

**Authors:** Hung-Wei Wang, Hsueh-Chou Lai, Wei-Fan Hsu, Sheng-Hung Chen, Yi-Chun Kuo, Cheng-Yuan Peng

**Affiliations:** a School of Medicine, China Medical University, Taichung, Taiwan; b Center for Digestive Medicine, Department of Internal Medicine, China Medical University Hospital, Taichung, Taiwan; c Graduate Institute of Biomedical Sciences, China Medical University, Taichung, Taiwan; d School of Chinese Medicine, China Medical University, Taichung, Taiwan

**Keywords:** Chronic hepatitis B, hepatocellular carcinoma, steatotic liver disease, cardiometabolic risk factor, nucleos(t)ide analog

## Abstract

**Background:**

Chronic hepatitis B (CHB) is a major cause of hepatocellular carcinoma (HCC). In patients receiving CHB treatment, coexistence of steatotic liver disease (SLD) and cardiometabolic risk factors (CMRFs) markedly influence HCC risk. This study explored hepatic and metabolic profiles, HCC predictors, and the modifying roles of SLD and CMRFs in CHB.

**Methods:**

This study included 1012 patients receiving nucleos(t)ide analogs between 2004 and 2022. They were stratified into SLD (*N* = 702) or non-SLD (*N* = 310) groups.

**Results:**

Over a 5.5-year follow-up period, 73 patients developed HCC. At baseline, the SLD group had younger age, higher body mass index values, lower fibrosis indices, and more severe metabolic dysregulation than did the non-SLD group. SLD conferred protection against HCC (adjusted hazard ratio: 0.43; *p* = 0.001). However, the presence of ≥2 CMRFs significantly increased HCC risk (adjusted hazard ratio: 1.93; *p* = 0.009). The highest HCC risk was observed in patients without SLD having ≥2 CMRFs (5-year incidence rate: 21.4%). The protective effect of SLD persisted after inverse probability of treatment weighting. It was most pronounced in older, cirrhotic, and high-metabolic-risk subgroups. Hepatitis B virus DNA suppression, hepatitis B surface antigen decline, and fibrosis score improvement during treatment were similar between the two groups.

**Conclusions:**

Among patients with CHB receiving nucleos(t)ide analogs, those without SLD having ≥2 CMRFs had the highest risk of HCC. Thus, steatosis and metabolic burden should be incorporated into precision risk stratification.

## KEY MESSAGES

- In this nucleos(t)ide analogue–treated chronic hepatitis B cohort (unlike prior studies that mixed treated and untreated patients), steatotic liver disease (SLD) was independently associated with a lower risk of hepatocellular carcinoma (HCC).
- HBV DNA suppression, hepatitis B surface antigen (HBsAg) decline, and fibrosis score improvement during therapy were similar between groups, suggesting that the lower HCC risk observed in patients with SLD is unlikely to be mediated by differential antiviral efficacy or enhanced HBV viral suppression.
- Cardiometabolic burden remained a major determinant of HCC risk during treatment, with the highest incidence observed in patients without SLD who had multiple cardiometabolic risk factors, while the biological mechanism linking SLD to reduced HCC risk remains unclear.

## Introduction

Chronic hepatitis B (CHB) is a major global health challenge, affecting more than 250 million individuals worldwide and contributing substantially to cirrhosis and hepatocellular carcinoma (HCC)–related morbidity and mortality [1,2]. Although potent nucleos(t)ide analogs (NAs) effectively suppress viral replication and improve long-term outcomes, a residual risk of HCC persists, indicating a need to identify nonviral risk factors for hepatocarcinogenesis [1]. Traditional risk factors for HCC include older age, male sex, advanced fibrosis, and cirrhosis. However, metabolic factors and hepatic steatosis have been gaining attention as modifiers of disease progression [1,3].

The terminology used to classify fatty liver disease has evolved from *nonalcoholic fatty liver disease* [4] and *metabolic dysfunction–associated fatty liver disease* (MAFLD) [5] to the broader term *steatotic liver disease* (SLD) [6], which encompasses multifactorial etiologies, such as viral hepatitis, alcohol consumption, and metabolic dysfunction. Hepatic steatosis is common among patients with CHB, given the increasing prevalence of obesity, diabetes, and dyslipidemia in this population. Zhang et al. [7] highlighted the paradoxical effects of hepatic steatosis on HCC risk. Hepatic steatosis is associated with a reduced content of hepatitis B virus (HBV) DNA, a low level of hepatitis B e antigen (HBeAg) expression, and a high rate of hepatitis B surface antigen (HBsAg) seroclearance; therefore, the presence of this condition inhibits HBV replication through mechanisms such as endoplasmic reticulum stress and adiponectin-mediated modulation. However, when accompanied by metabolic dysfunction, hepatic steatosis exacerbates inflammation and fibrosis in the liver, thereby increasing the risk of HCC [7]. Large-scale cohort and population-based studies have confirmed this duality [8–11]. In patients with CHB, isolated hepatic steatosis reduces the risks of HCC and all-cause mortality, exerting protective effects [11]. However, coexistence of hepatic steatosis with cardiometabolic risk factors (CMRFs), such as diabetes mellitus (DM), obesity, hypertension, and dyslipidemia, substantially increases the risks of HCC and mortality [11]. Despite these findings, the interaction effects of SLD and CMRFs on HCC risk in patients with CHB receiving antiviral therapy remains underexplored. Accordingly, the present study (1) compared baseline hepatic and metabolic profiles between patients with CHB with SLD and those without SLD, (2) identified independent predictors of HCC incidence during antiviral therapy, (3) determined whether SLD modifies virological and serological responses to NA treatment, and (4) estimated the HCC risk conferred by the concomitant presence of multiple CMRFs and SLD. The findings may guide precision risk stratification and help prevent CHB-related HCC.

## Methods

### Study cohort

**Figure 1.**
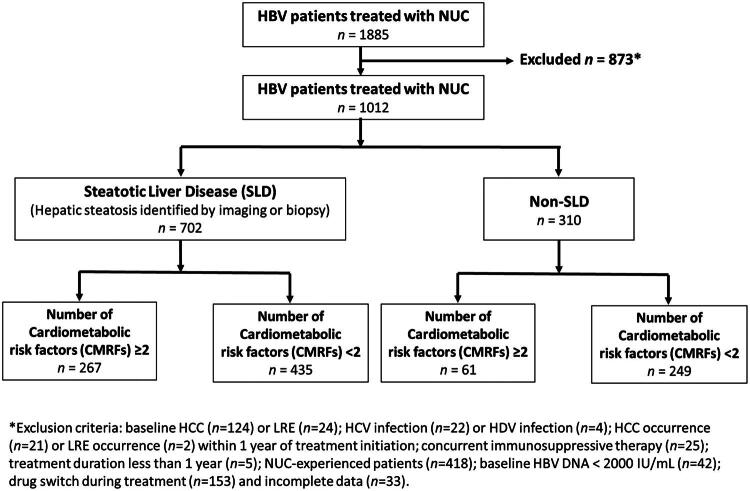
Study flow diagram for cohort selection and subgroup stratification. HBV: hepatitis B virus; NUC: nucleos(t)ide analogue; SLD: steatotic liver disease; CMRF: cardiometabolic risk factor; HCC: hepatocellular carcinoma; LRE: liver-related event; HCV: hepatitis C virus; HDV: hepatitis D virus.

A total of 1885 patients who had received a CHB diagnosis between 2004 and 2022 at our hospital were screened on the basis of the availability of serial clinical and laboratory data. The inclusion criteria were as follows: having a confirmed CHB diagnosis, receiving regular antiviral therapy, and being monitored for >12 months. The exclusion criteria were as follows: receiving an HCC diagnosis at baseline or within 1 year after treatment initiation; undergoing liver transplantation; having coinfection with hepatitis C virus, hepatitis D virus, or HIV; and having insufficient data (Figure 1). Patients with known competing causes of chronic liver disease identified in the clinical record, including other established non-HBV etiologies, were excluded from the analytic cohort. However, systematic screening for occult autoimmune liver disease was not uniformly available in this retrospective study.

Eligible patients constituted the final cohort. The patients were stratified by SLD status into SLD and non-SLD groups. SLD was diagnosed through abdominal ultrasonography or biopsy. Cirrhosis was diagnosed by ultrasonography or biopsy, with biopsy available only in a minority. Alcohol consumption was defined as a daily consumption amount of >20 g in women and >30 g in men. HCC was diagnosed on the basis of the American Association for the Study of Liver Diseases or European Association for the Study of the Liver criteria [12,13]. A definite diagnosis of HCC was confirmed through noninvasive multiphasic computed tomography, magnetic resonance imaging, or histopathological examination.

### CMRFs

CMRFs were defined as per established clinical criteria [6]. Obesity in our Asian cohort was defined by a body mass index (BMI) of ≥23 kg/m^2^ or by an elevated waist circumference of >94 cm in men or >80 cm in women. Dysglycemia was defined as a fasting plasma glucose level of ≥100 mg/dL, a glycated hemoglobin percentage of ≥5.7%, or the use of drugs for type 2 DM. Hypertension was defined as systolic/diastolic blood pressure of ≥130/85 mmHg or the use of antihypertensive medication. Dyslipidemia was defined as a triglyceride level of ≥150 mg/dL, a high-density lipoprotein cholesterol (HDL-C) level of ≤40 mg/dL in men or ≤50 mg/dL in women, or the use of lipid-lowering drugs.

### Ethical considerations

This study adhered to the ethical principles of the 1975 Declaration of Helsinki. The requirement for informed consent was waived because patient identification numbers were anonymized in this retrospective study, which ensured confidentiality. The study protocol was approved by the Research Ethics Committee of China Medical University Hospital, Taiwan (approval number: CMUH102-REC1-113).

### Statistical analysis

Nonnormally distributed continuous variables are presented as median (interquartile range) values, whereas categorical variables are presented as frequency (percentage) values. Continuous variables were compared using the Mann–Whitney U test, whereas categorical variables were compared using the chi-square test or Fisher’s exact test. Cox regression was performed to identify factors associated with HCC incidence. Inverse probability of treatment weighting (IPTW) was performed to minimize confounding effects in propensity score estimation; the model was adjusted for age, cirrhosis status, and fibrosis-4 index (FIB-4) score. Cumulative HCC incidence was evaluated using the Kaplan–Meier method and a log-rank test. Subgroup hazard ratios (HRs) with corresponding 95% confidence intervals (CIs) were calculated, and forest plots were constructed to depict the effects of SLD across subgroups of patients with CHB receiving NAs. Analyses were performed using SPSS (version 25.0) and Stata/SE (version 16.1). A two-tailed *p* value of <0.05 indicated significance.

## Results

### Hepatic and metabolic profiles of the SLD and non-SLD groups

This study included 1012 patients with CHB who had received oral antiviral therapy. The SLD and non-SLD groups comprised 702 and 310 patients, respectively. Over a median treatment duration of 5.5 years, 73 patients developed HCC. At baseline, the SLD group was younger than the non-SLD group (48 vs. 50 years; *p* = 0.032). The SLD group had higher BMI values than did the non-SLD group (24.7 vs. 23.0 kg/m^2^; *p* < 0.001). Furthermore, the SLD group had a significantly lower cirrhosis prevalence, higher platelet counts, and lower FIB-4 scores than did the non-SLD group, indicating less advanced fibrosis. Laboratory assessments revealed lower aspartate transaminase (AST) levels in the SLD group than in the non-SLD group. However, the groups had similar alanine transaminase (ALT) levels. HDL-C levels were lower and triglyceride levels were higher in the SLD group than in the non-SLD group (*p* < 0.001 and *p* = 0.009, respectively), consistent with metabolic dysregulation. The two groups were similar in terms of HBV DNA content, quantitative HBsAg (qHBsAg) level, and HBeAg status. However, the incidence of HCC was significantly lower in the SLD group than in the non-SLD group (39/702 [5.6%] vs. 34/310 [11.0%]; *p* = 0.003; Table 1). These findings indicate that the two groups had distinct metabolic and hepatic profiles at treatment initiation and different outcomes during long-term treatment.

**Table 1.** Baseline clinicodemographic characteristics of patients with CHB receiving antiviral therapy (*N* = 1012).

Parameter n (%) or median [IQR]	Sample number	CHB without SLD (*N* = 310)	CHB with SLD (*N* = 702)	*p* value
Age, year	1012	50 [20]	48 [15]	0.032
Male	1012	208 (67.1)	499 (71.1)	0.207
Alcohol	1012	57 (18.4)	142 (20.2)	0.548
BMI, kg/m^2^	914	23.0 [3.8]	24.7 [4.4]	<0.001
DM	1012	49 (15.8)	108 (15.4)	0.851
Pre-DM and DM	1012	80 (25.8)	221 (31.5)	0.074
Hypertension	1012	49 (15.8)	141 (20.1)	0.116
Cirrhosis*	1012	128 (41.3)	184 (26.2)	<0.001
qHBsAg, log IU/mL	938	3.17 [0.87]	3.27 [0.93]	0.064
HBeAg, positivity	1012	106 (34.2)	259 (36.9)	0.435
HBV DNA, log IU/mL	1012	6.05 [2.41]	6.16 [2.51]	0.572
AST, U/L	1005	77 [192]	66 [96.5]	0.002
ALT, U/L	1012	96 [273]	104.5 [194.3]	0.983
Platelet count,10^9^/L	977	153 [89]	174 [73.8]	<0.001
AFP, ng/mL	968	5.95 [18.8]	4.81 [7.09]	<0.001
FIB-4	972	3.17 [4.90]	1.94 [2.00]	<0.001
AC Glucose, mg/dL	581	96 [16.8]	97 [15]	0.192
TG^1^, mg/dL	667	86.5 [47.3]	96 [62]	0.009
HDL-C^2^, mg/dL	507	55.6 [23.5]	48.1 [17.8]	<0.001
ETV/TDF/TAF/Others	1012	246/50/6/8	540/123/25/14	0.457
Treatment duration, year	1012	5.10 [5.23]	5.80 [6.00]	0.247
HCC incidence	1012	34 (11.0)	39 (5.6)	0.003

^1^ A plasma TG level of ≥150 mg/dL or the use of lipid-lowering drugs.

^2^ A plasma HDL-C level of ≤40 mg/dL in men or ≤50 mg/dL in women or the use of lipid-lowering drugs.

* Cirrhosis was diagnosed through ultrasonography or liver biopsy (*N* = 118).

AC glucose: fasting (*ante cibum*) glucose; AFP: alpha-fetoprotein; ALT: alanine transaminase; AST: aspartate transaminase; BMI: body mass index; CHB: chronic hepatitis B; DM: diabetes mellitus; FIB-4: fibrosis-4 index; HBeAg: hepatitis B e antigen; HBV: hepatitis B virus; HCC: hepatocellular carcinoma; HDL-C: high-density lipoprotein cholesterol; IQR: interquartile range; IU: international unit; Pre-DM: prediabetes; qHBsAg: quantitative hepatitis B surface antigen; SLD: steatotic liver disease; TG: triglyceride.

### Risk factors for HCC in patients receiving CHB treatment

**Figure 2.**
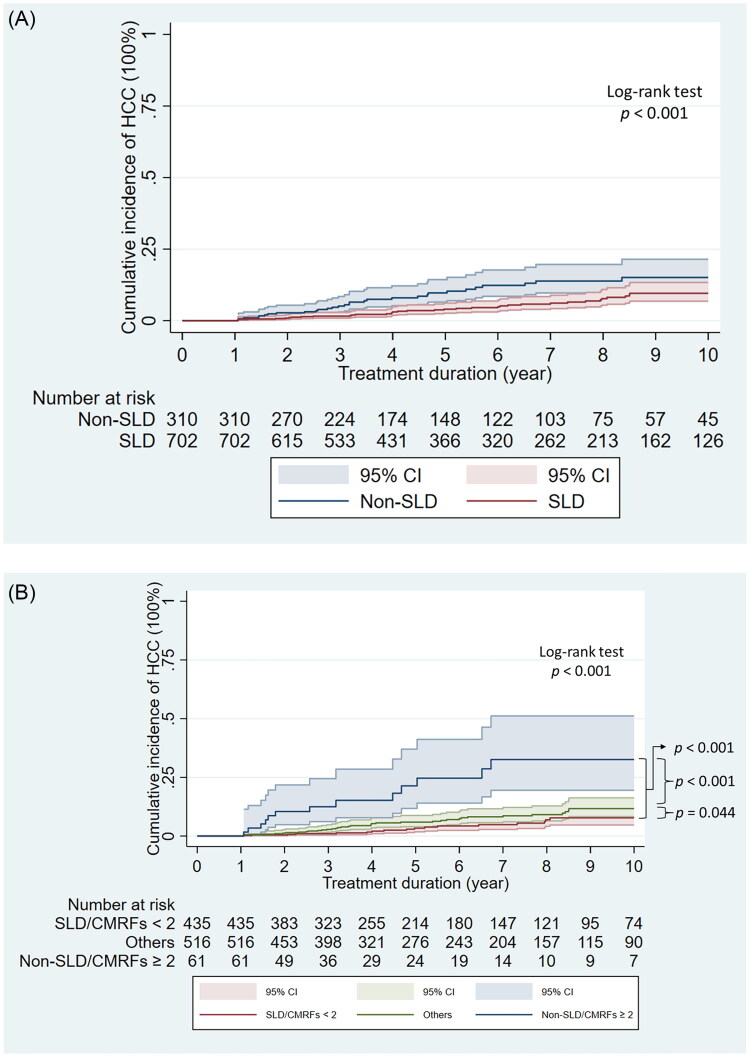
Cumulative incidence of HCC during nucleos(t)ide analogue therapy. (A) Kaplan–Meier curves comparing patients with SLD versus those without SLD (non-SLD). (B) Kaplan–Meier curves stratified by combined SLD status and CMRF burden: SLD with <2 CMRFs, non-SLD with ≥2 CMRFs, and ‘Others’ (SLD with ≥2 CMRFs or non-SLD with <2 CMRFs). HCC: hepatocellular carcinoma; SLD: steatotic liver disease; CMRF: cardiometabolic risk factor; CI: confidence interval.

Tables 2 and 3 present the risk factors for HCC in patients with CHB receiving antiviral therapy. SLD conferred protection against HCC (adjusted HR [aHR]: 0.431; *p* = 0.001; Table 2; Figure 2A). An FIB-4 score of ≥3.25 emerged as an independent risk factor. The presence of ≥2 CMRFs, but not ≥1 CMRF, was independently associated with a significantly increased risk of HCC (aHR: 1.927; *p* = 0.009). Among the five CMRFs, DM and pre-DM/DM exerted the largest effect on HCC occurrence (HR ≈ 2.0 and 1.8, respectively). Elevated BMI, hypertension, hypertriglyceridemia, and low HDL-C level were associated with relatively modest and statistically nonsignificant increases in HCC risk (Supplementary Tables 1 and 2).

**Table 2.** Factors associated with HCC incidence in the cardiometabolic risk factor–based model (*N* = 1012).

Variables	Crude Hazard Ratio (95% CI)	*p* value	Adjusted Hazard Ratio (95% CI)	*p* value
Age ≥50 vs. <50 (year)	3.637 (2.136–6.193)	<0.001		
Gender, male vs. female	1.073 (0.641–1.795)	0.788		
Alcohol	1.754 (1.056–2.912)	0.030	1.671 (0.997–2.801)	0.052
SLD	0.467 (0.295–0.739)	0.001	0.431 (0.261–0.712)	0.001
BMI ≥23 vs. <23 (kg/m^2^]	1.567 (0.900–2.731)	0.113		
DM	2.054 (1.217–3.465)	0.007		
Pre-DM and DM	1.804 (1.136–2.864)	0.012		
Hypertension	1.375 (0.821–2.302)	0.226		
TG^1^, mg/dL	0.926 (0.289–2.968)	0.897		
HDL-C^2^, mg/dL	0.999 (0.352–2.833)	0.998		
Number of cardiometabolic risk factors, CMRFs, n ≥1 vs. n <1	1.732 (0.912–3.290)	0.093		
Number of cardiometabolic risk factors, CMRFs, n ≥2 vs. n <2	1.647 (1.038–2.612)	0.034	1.927 (1.177–3.154)	0.009
HBeAg, positivity	0.782 (0.477–1.281)	0.329		
AST (per U/L increase)	0.997 (0.994–0.999)	0.006		
ALT (per U/L increase)	0.997 (0.994–0.999)	0.001		
PLT count (per 10^9^/L increase)	0.987 (0.982–0.991)	0.001		
AFP ≥9 vs. <9 (ng/mL)	1.347 (0.835–2.172)	0.222		
FIB-4 ≥ 3.25 vs. <3.25	2.389 (1.504–3.795)	<0.001	1.929 (1.195–3.113)	0.007

^1^ A plasma TG level of ≥150 mg/dL or the use of lipid-lowering drugs.

^2^ A plasma HDL-C level of ≤40 mg/dL in men and ≤50 mg/dL in women or the use of lipid-lowering drugs.

Shaded cells indicate that the variable exerted a confounding effect on other factors and was therefore not included in the multivariate model.

AFP: alpha-fetoprotein; ALT: alanine transaminase; AST: aspartate transaminase; BMI: body mass index; CI: confidence interval; CMRF: cardiometabolic risk factor; DM: diabetes mellitus; FIB-4: fibrosis-4 index; HBeAg: hepatitis B e antigen; HDL-C: high-density lipoprotein cholesterol; HCC: hepatocellular carcinoma; PLT: platelet; Pre-DM: prediabetes; SLD: steatotic liver disease; TG: triglyceride.

**Table 3.** Factors associated with HCC incidence in the combined SLD and cardiometabolic risk factor model (*N* = 1012).

Variables	Crude Hazard Ratio (95% CI)	*p* value	Adjusted Hazard Ratio (95% CI)	*p* value
Age ≥50 vs. <50 (year)	3.637 (2.136–6.193)	<0.001		
Gender, male vs. female	1.073 (0.641–1.795)	0.788		
Alcohol	1.754 (1.056–2.912)	0.030	1.703 (1.015–2.857)	0.044
SLD	0.467 (0.295–0.739)	0.001		
BMI ≥23 vs. <23 (kg/m^2^]	1.567 (0.900–2.731)	0.113		
DM	2.054 (1.217–3.465)	0.007		
Pre-DM and DM	1.804 (1.136–2.864)	0.012		
Hypertension	1.375 (0.821–2.302)	0.226		
TG^1^	0.926 (0.289–2.968)	0.897		
HDL-C^2^	0.999 (0.352–2.833)	0.998		
Number of cardiometabolic risk factor, CMRFs, n ≥1 vs. n <1	1.732 (0.912–3.290)	0.093		
Number of cardiometabolic risk factor, CMRFs, n ≥2 vs. n <2	1.647 (1.038–2.612)	0.034		
Combination of SLD and CMRF				
SLD (+) & CMRFs n <2	1		1	
SLD (+) & CMRFs n ≥2; SLD (-) & CMRFs n <2	1.738 (1.009–2.995)	0.046	1.480 (0.850–2.578)	0.166
SLD (-) & CMRFs n ≥2	6.063 (2.992–12.29)	<0.001	4.943 (2.379–10.27)	<0.001
HBeAg, positivity	0.782 (0.477–1.281)	0.329		
AST (per U/L increase)	0.997 (0.994–0.999)	0.006		
ALT (per U/L increase)	0.997 (0.994–0.999)	0.001		
PLT count (per 10^9^/L increase)	0.987 (0.982–0.991)	0.001		
AFP ≥9 vs. <9 (ng/mL)	1.347 (0.835–2.172)	0.222		
FIB-4 ≥ 3.25 vs. <3.25	2.389 (1.504–3.795)	<0.001	1.962 (1.215–3.169)	0.006

^1^ A plasma TG level of ≥150 mg/dL or the use of lipid-lowering drugs.

^2^ A plasma HDL-C level of ≤40 mg/dL in men and ≤50 mg/dL in women or the use of lipid-lowering drugs.

Shaded cells indicate that the variable exerted a confounding effect on other factors and was therefore not included in the multivariate model.

AFP, alpha-fetoprotein; ALT: alanine transaminase; AST: aspartate transaminase; BMI: body mass index; CI: confidence interval; CMRF: cardiometabolic risk factor; DM: diabetes mellitus; FIB-4: fibrosis-4 index; HBeAg: hepatitis B e antigen; HDL-C: high-density lipoprotein cholesterol; HCC: hepatocellular carcinoma; PLT: platelet; Pre-DM: prediabetes; SLD: steatotic liver disease; TG: triglyceride.

Consumption of alcohol and an FIB-4 score of ≥3.25 were independently associated with HCC risk. Patients without SLD having ≥2 CMRFs exhibited a markedly elevated risk of HCC (aHR: 4.943; *p* < 0.001; Table 3), whereas those with SLD exhibited a reduced risk. Notably, HCC risk was approximately five-fold higher in patients without SLD having ≥2 CMRFs than in those with SLD having <2 CMRFs (Figure 2B). Furthermore, the 5-year cumulative incidence rate of HCC was markedly higher in patients without SLD having ≥2 CMRFs than in those with SLD having <2 CMRFs (21.4% vs. 3.4%), underscoring the synergistic effect of metabolic burden and no steatosis on HCC incidence.

### Independent predictors of HCC after IPTW

Before IPTW, the SLD group was younger than the non-SLD group. Furthermore, the SLD group had higher BMI values, lower cirrhosis prevalence, lower FIB-4 scores, higher platelet counts, and more severe metabolic dysregulation (including higher triglyceride and lower HDL-C levels) than did the non-SLD group. The levels of AST and ALT were significantly lower in the SLD group than in the non-SLD group.

After IPTW, baseline characteristics were well balanced between the two groups, with no significant differences in demographics, viral markers, liver function, or fibrosis indicators (Table 4). Even after IPTW, several factors remained significantly associated with HCC incidence (Table 5). The incidence of HCC remained significantly lower in the SLD group than in the non-SLD group, highlighting consistent protective effects of SLD against HCC (aHR: 0.461; 95% CI: 0.250–0.848; *p* = 0.013). However, the presence of ≥2 CMRFs was associated with an increased risk of HCC (aHR: 2.093; 95% CI: 1.078–4.064; *p* = 0.029). A borderline association was observed between HCC incidence and advanced fibrosis, defined as an FIB-4 score of ≥3.25 (aHR: 1.853; 95% CI: 0.998–3.439; *p* = 0.051). Other predictors such as alcohol consumption and HBeAg positivity became nonsignificant after IPTW.

**Table 4.** Baseline clinicodemographic characteristics of the study cohort before and after IPTW.

	Before IPTW			After IPTW		
Parameter n (%) or mean [SD]	CHB without SLD (*N* = 310)	CHB with SLD (*N* = 702)	*p* value	CHB without SLD (*N* = 805)	CHB with SLD (*N* = 846)	*p* value
Age, year	50.1 [13.6]	48.1 [10.9]	0.014	48.9 [13.7]	48.9 [10.9]	0.926
Male	208 (67.1)	499 (71.1)	0.207	558 (69.3)	586 (69.3)	>0.999
Alcohol	57 (18.4)	142 (20.2)	0.548	149 (18.5)	166 (19.6)	0.573
BMI, kg/m^2^	23.3 [3.4]	25.1 [3.5]	<0.001	24.4 [3.8]	24.6 [3.5]	0.404
DM	49 (15.8)	108 (15.4)	0.925	129 (16)	126 (14.9)	0.540
Pre-DM and DM	80 (25.8)	221 (31.5)	0.074	229 (28.4)	247 (29.2)	0.745
Hypertension	49 (15.8)	141 (20.1)	0.116	160 (19.9)	179 (21.2)	0.543
Cirrhosis*	128 (41.3)	184 (26.2)	<0.001	258 (32)	268 (31.7)	0.874
qHBsAg, log IU/mL	3.14 [0.88]	3.24 [0.87]	0.108	3.19 [0.93]	3.23 [0.87]	0.414
HBeAg, positivity	106 (34.2)	259 (36.9)	0.435	277 (34.4)	306 (36.2)	0.471
HBV DNA, log IU/mL	6.15 [1.57]	6.18 [1.50]	0.827	6.22 [1.58]	6.19 [1.48]	0.745
AST, U/L	226.9 [329.6]	168.8 [282.4]	0.004	195.3 [274.1]	183.7 [293.2]	0.405
ALT, U/L	320.4 [485.1]	260.9 [392.0]	0.039	305.8 [434.5]	260.5 [375.4]	0.023
Platelet count, 10^9^/L	155.7 [64.4]	176.1 [57.4]	<0.001	168.7 [64.0]	169.8 [56.8]	0.706
AFP, ng/mL	35.0 [86.4]	31.8 [168.5]	0.758	35.0 [89.6]	34.2 [174.7]	0.908
FIB-4	4.89 [5.50]	2.99 [3.36]	<0.001	3.66 [3.78]	3.53 [4.46]	0.508
AC Glucose, mg/dL	106.2 [35.4]	105.8 [34.0]	0.902	102.5 [27.7]	106.1 [38.8]	0.120
TG^1^, mg/dL	95.2 [49.5]	108.4 [62.0]	0.010	90.5 [41.1]	106.3 [59.8]	<0.001
HDL-C^2^, mg/dL	57.1 [15.9]	50.5 [14.4]	<0.001	57.2 [13.9]	51.7 [15.1]	<0.001
HCC incidence	34 (11)	39 (5.6)	0.003	81 (10.1)	46 (5.4)	<0.001

Study cohort: patients with CHB receiving antiviral therapy.

^1^ A plasma TG level of ≥150 mg/dL or the use of lipid-lowering drugs.

^2^ A plasma HDL-C level of ≤40 mg/dL in men or ≤50 mg/dL in women or the use of lipid-lowering drugs.

* Cirrhosis was diagnosed through ultrasonography or liver biopsy (*N* = 118).

AC glucose: fasting (*ante cibum*) glucose; AFP: alpha-fetoprotein; ALT: alanine transaminase; AST: aspartate transaminase; BMI: body mass index; CHB: chronic hepatitis B; DM: diabetes mellitus; FIB-4: fibrosis-4 index; HBeAg: hepatitis B e antigen; HBV: hepatitis B virus; HDL-C: high-density lipoprotein cholesterol; HCC: hepatocellular carcinoma; IPTW: inverse probability of treatment weighting; IU: international unit; PLT: platelet; Pre-DM: prediabetes; qHBsAg: quantitative hepatitis B surface antigen; SD: standard deviation; SLD: steatotic liver disease; TG: triglyceride.

**Table 5.** Factors associated with HCC incidence in the cardiometabolic risk factor–based model after IPTW.

Variables	Crude Hazard Ratio (95% CI)	*p* value	Adjusted Hazard Ratio (95% CI)	*p* value
Age ≥50 vs. <50 (year)	3.862 (2.030–7.347)	<0.001		
Gender, female vs male	0.827 (0.422–1.622)	0.581		
Alcohol	1.833 (0.983–3.416)	0.057	1.727 (0.870–3.429)	0.118
SLD	0.511 (0.294–0.889)	0.017	0.461 (0.250–0.848)	0.013
BMI ≥23 vs. <23 (kg/m^2^]	1.732 (0.897–3.342)	0.102		
DM	2.229 (1.112–4.467)	0.024		
Pre-DM and DM	2.066 (1.132–3.769)	0.018		
Hypertension	1.851 (0.963–3.557)	0.065		
TG^1^, mg/dL	0.379 (0.051–2.824)	0.344		
HDL-C^2^, mg/dL	1.640 (0.324–8.295)	0.550		
Number of cardiometabolic risk factor, CMRFs, *n* ≥ 1 vs. *n* < 1	2.984 (1.311–6.794)	0.009		
Number of cardiometabolic risk factor, CMRFs, *n* ≥ 2 vs. *n* < 2	2.112 (1.172–3.806)	0.013	2.093 (1.078–4.064)	0.029
HBeAg, positivity	0.607 (0.340–1.082)	0.090	0.742 (0.400–1.374)	0.342
AST (per U/L increase)	0.996 (0.994–0.998)	0.003		
ALT (per U/L increase)	0.996 (0.994–0.999)	0.010		
PLT count (per 10^9^/L increase)	0.989 (0.983–0.995)	<0.001		
AFP ≥9 vs. <9 (ng/mL)	1.267 (0.688–2.332)	0.447		
FIB-4 ≥ 3.25 vs. <3.25	2.232 (1.230–4.053)	0.008	1.853 (0.998–3.439)	0.051

^1^ A plasma TG level of ≥150 mg/dL or the use of lipid-lowering drugs.

^2^ A plasma HDL-C level of ≤40 mg/dL in men and ≤50 mg/dL in women or the use of lipid-lowering drugs.

Shaded cells indicate that the variable exerted a confounding effect on other factors and was therefore not included in the multivariate model.

AFP: alpha-fetoprotein; ALT: alanine transaminase; AST: aspartate transaminase; BMI: body mass index; CI: confidence interval; CMRF: cardiometabolic risk factor; DM: diabetes mellitus; FIB-4: fibrosis-4 index; HBeAg: hepatitis B e antigen; HDL-C: high-density lipoprotein cholesterol; HCC: hepatocellular carcinoma; IPTW: inverse probability of treatment weighting; PLT: platelet; Pre-DM: prediabetes; SLD: steatotic liver disease; TG: triglyceride.

### Results of subgroup analyses of HCC risk in the SLD and non-SLD groups

**Figure 3.**
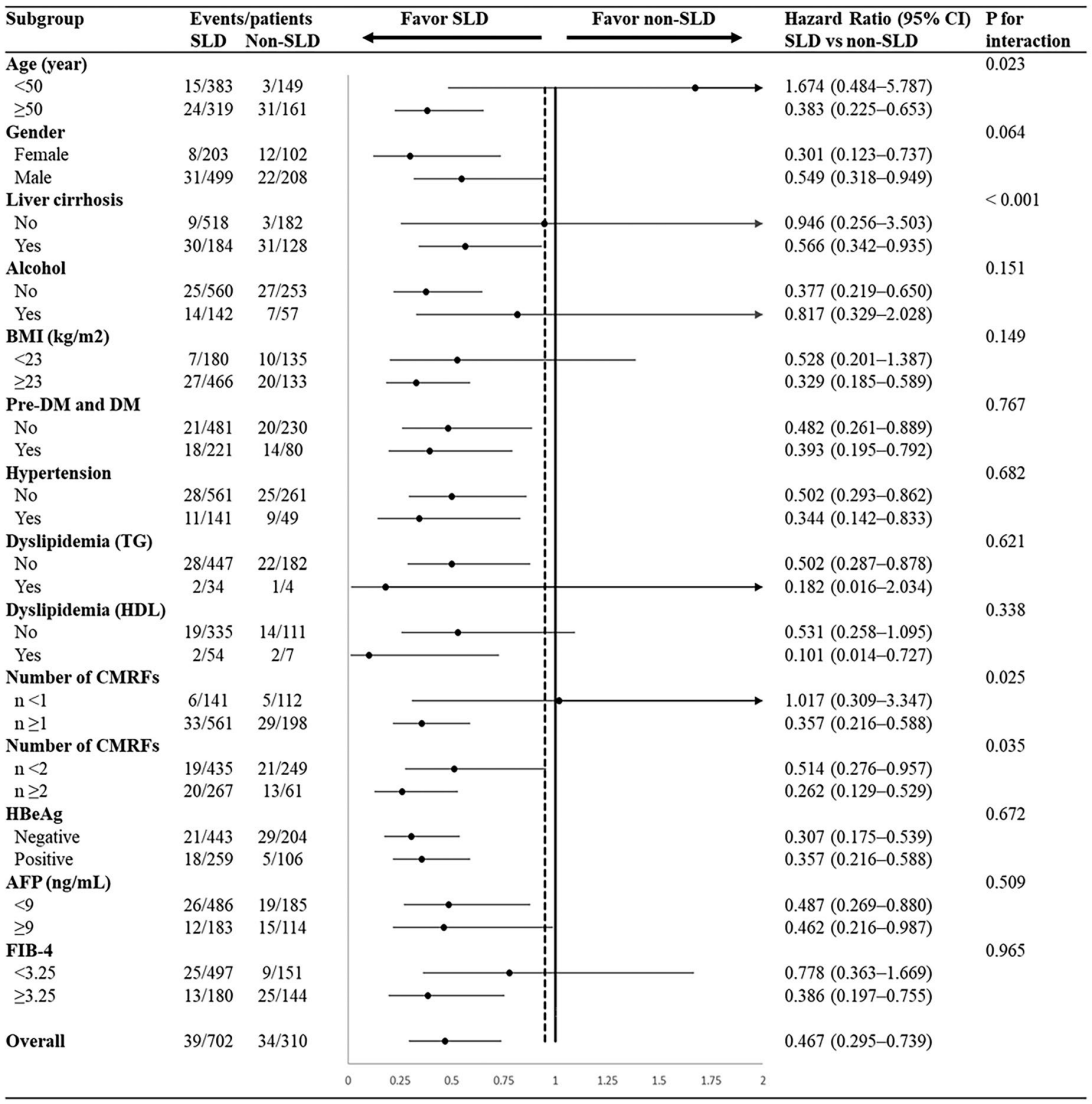
Subgroup analysis of HCC risk associated with SLD during nucleos(t)ide analogue therapy. SLD: steatotic liver disease; CMRF: cardiometabolic risk factor; HR: hazard ratio; CI: confidence interval; BMI: body mass index; Pre-DM: prediabetes; DM: diabetes mellitus; TG: triglycerides; HDL: high-density lipoprotein; HBeAg: hepatitis B e antigen; AFP: alpha-fetoprotein.

SLD was associated with a reduced risk of HCC (HR: 0.467; 95% CI: 0.295–0.739; Figure 3). The protective effect of SLD was most pronounced in older patients (≥50 years; HR: 0.383; 95% CI: 0.225–0.653; *p* for interaction =0.023) and those with liver cirrhosis (HR: 0.566; 95% CI: 0.342–0.935; *p* for interaction < 0.001). The protective effect varied also by CMRF count. The HR was 0.357 (95% CI: 0.216–0.588) for ≥1 CMRF (1–5 risk factors), 0.262 (95% CI: 0.129–0.529) for ≥2 CMRFs (2–5 risk factors), and 0.514 (95% CI: 0.276–0.957) for <2 CMRFs (0–1 risk factor). Patients with ≥2 CMRFs exhibited a marked increase in HCC risk (*p* for interaction =0.035). These findings suggest that SLD confers protection against HCC in patients with CHB, particularly those with high metabolic risks or cirrhosis.

### Effect of SLD on HCC risk in patients with CHB having multiple CMRFs

Among patients with CHB having ≥2 CMRFs (*N* = 328), those with SLD (*N* = 267) exhibited more favorable hepatic profiles than did those without SLD. The SLD group was younger than the non-SLD group (median age: 52 vs. 60 years; *p* < 0.001). Furthermore, the SLD group had higher BMI values than did the non-SLD group (25.6 vs. 24.5 kg/m^2^; *p* = 0.020). The prevalence of cirrhosis was lower in the SLD group than in the non-SLD group (86/267 [32.2%] vs. 35/61 [57.4%]; *p* < 0.001). FIB-4 scores were lower in the SLD group than in the non-SLD group (2.13 vs. 3.90; *p* < 0.001). However, platelet counts were higher in the SLD group than in the non-SLD group (169 vs. 142 × 10^9^/L; *p* = 0.002). These findings indicated that the severity of fibrosis was lower in the SLD group than in the non-SLD group. Laboratory data indicated lower AST levels (*p* = 0.039), alpha-fetoprotein levels (*p* = 0.009), and HDL-C levels (*p* = 0.034) in the SLD group than in the non-SLD group. HCC incidence was significantly lower in the SLD group than in the non-SLD group (20/267 [7.49%] vs. 13/61 [21.3%]; *p* = 0.003; Supplementary Table 3). In CHB patients with multiple cardiometabolic risk factors, HCC cumulative incidence was significantly higher in those without SLD than in those with SLD (log-rank *p* < 0.001; Supplementary Figure 1).

Multivariate Cox regression confirmed SLD as an independent protective factor against HCC (aHR: 0.262; 95% CI 0.125–0.548; *p* < 0.001). Advanced fibrosis (FIB-4 score ≥3.25) exhibited only a borderline crude association with HCC incidence (HR: 1.833; *p* = 0.087), and this association lost significance after IPTW (Supplementary Table 4). After IPTW, baseline imbalance was reduced but not fully eliminated in the ≥2 CMRFs subgroup, with residual differences observed for BMI, hypertension, ALT, and HDL-C (Supplementary Table 5). The protective effect of SLD remained consistent (7.5% vs. 21.3% [*p* = 0.003] 8.0% vs. 18.6% [*p* < 0.001]). Overall, SLD conferred robust protection against HCC in patients with CHB having ≥2 CMRFs (aHR: 0.382; 95% CI 0.172–0.852; *p* = 0.019; Supplementary Table 6).

### Longitudinal changes in FIB-4, liver stiffness measurement, qHBsAg, and HBV DNA in the SLD and non-SLD groups

Across 5 years of therapy, on-treatment virologic responses were comparable by SLD status, with similar declines in qHBsAg (*p* = 0.727) and sustained HBV DNA suppression (*p* = 0.595). Fibrosis surrogates improved in both groups; FIB-4 decreased but remained higher in the non-SLD group (*p* = 0.045), whereas liver stiffness declined without between-group differences (*p* = 0.721) (Supplementary Figure 2A–D).

In older patients, fibrosis severity was consistently lower in the SLD group than in the non-SLD group at baseline (median FIB-4 score: 2.45 vs. 4.42; *p* < 0.001) and at years 1, 3, and 5 (Supplementary Table 7). In addition, liver stiffness measurements (LSMs) were lower in the SLD group than in the non-SLD group at year 1 (median LSM: 1.20 vs. 1.38 m/s; *p* = 0.002) and remained lower throughout follow-up. In patients with cirrhosis, baseline FIB-4 scores were markedly lower in the SLD group than in the non-SLD group (median score: 2.38 vs. 5.31; *p* < 0.001); this difference persisted over time. In patients with ≥1, ≥2, or <2 CMRFs, baseline FIB-4 scores were consistently and significantly lower in the SLD group than in the non-SLD group (median score: 1.95 vs. 3.89, 2.13 vs. 3.90, and 1.84 vs. 3.00, respectively; all *p* < 0.001); this pattern persisted throughout follow-up. Similar trends were observed for LSM. In older patients, those with cirrhosis, and those with CMRFs, qHBsAg levels were higher in the SLD group than in the non-SLD group at most time points whereas HBV DNA contents were similar (Supplementary Table 8). Posttreatment percentage changes (from baseline) in FIB-4 score, LSM, qHBsAg level, and HBV DNA content did not significantly differ between the SLD and non-SLD groups (Supplementary Table 9).

## Discussion

In this large cohort of NA-treated patients with CHB, SLD was independently associated with a reduced risk of HCC, consistent with the literature [8,14–16]. This protective effect persisted after IPTW, underscoring its robustness. By contrast, the presence of ≥2 CMRFs independently predicted HCC, highlighting the opposing roles of steatosis and metabolic dysfunction in HBV-related carcinogenesis. Compared with patients without SLD, at treatment initiation, the SLD group exhibited more adverse metabolic profiles, lower fibrosis scores (FIB-4 and liver stiffness), higher platelet counts, and marginally higher qHBsAg levels than did the non-SLD group. However, the two groups exhibited similar HBV DNA suppression, qHBsAg decline, and fibrosis score improvement and the SLD group maintained low fibrosis severity during treatment (Supplementary Tables 8 and 9).

A matched meta-analysis involving 13,262 patients with CHB reported reduced HCC risk in those with steatosis [9]. Studies have revealed an increased risk of HCC in patients with CHB plus metabolic syndrome [17,18]. In the current study, we discovered that the protective effect of SLD was particularly evident in older patients (≥50 years), those with cirrhosis, and those with ≥2 CMRFs. Consistent with our study, a study involving patients with CHB receiving antiviral therapy reported that a controlled attenuation parameter value of ≥222 dB/m was associated with a significantly lower 5-year HCC incidence in patients with advanced chronic liver disease than in those without it (11.0% vs. 24.0%; *p* = 0.002) [16]. Huang et al. reported that MAFLD was associated with a reduced HCC risk and that hepatic steatosis and metabolic dysfunction exerted opposing effects on HCC incidence in treatment-naïve patients with CHB [19]. The protective effect of MAFLD was most evident in patients with elevated BMI (≥23 kg/m^2^), HBeAg negativity, no cirrhosis, and low to intermediate FIB-4 scores (≤3.25) [19]. These findings differ from those of our study, likely because Huang et al. used the composite definition of MAFLD. SLD and metabolic dysfunction should be assessed separately, given their opposing effects on HCC risk.

Mechanistically, hepatic steatosis may attenuate HBV replication through endoplasmic reticulum stress, adiponectin-mediated immunomodulation, and altered lipid metabolism, thereby exerting a protective effect [7]. By contrast, metabolic dysfunction promotes hepatocarcinogenesis through insulin resistance, lipotoxicity, and chronic low-grade inflammation, which accelerate fibrogenesis and facilitate malignant transformation [20,21]. These opposing mechanisms may partly explain the paradox wherein steatosis alone may confer protection, whereas its coexistence with multiple CMRFs markedly increases HCC risk. A study reported that steatosis alone promoted HBsAg seroclearance in treatment-naïve patients with CHB [22]. However, our study demonstrated that steatosis neither suppressed HBV DNA or qHBsAg before or during treatment nor accelerated fibrosis regression in the SLD group compared with in the non-SLD group. Imbalance in baseline fibrosis status ostensibly explained the between-group differences in HCC incidence. Nonetheless, steatosis remained an independent protective factor after IPTW. Thus, in patients with CHB whose disease activity necessitates antiviral therapy, steatosis confers protection through mechanisms that appear independent of HBV DNA or qHBsAg suppression or fibrosis regression [7]. Whether the underlying mechanisms involve altered immune microenvironment, epigenetic modifications, or dysbiosis independently of HBV replication remain to be elucidated [7,23,24].

In patients with CHB receiving NAs, SLD conferred protection against HCC, whereas the presence of CMRFs markedly increased HCC risk. DM emerged as the strongest predictor of HCC, and the presence of ≥2 CMRFs nearly doubled the risk of this cancer (Table 2 and Supplementary Tables 1 and 2). Notably, among patients with ≥2 CMRFs, those without SLD exhibited significantly lower platelet counts, higher FIB-4 scores, and higher HCC risks than did those with SLD (Supplementary Table 3). In the multivariate Cox regression analysis, SLD outperformed FIB-4 score in independently predicting HCC incidence (Supplementary Tables 4 and 6). Notably, patients without SLD having ≥2 CMRFs exhibited the highest risk of HCC during treatment. We offer two possible explanations for this finding. First, a multicenter study on MASLD demonstrated that patients with ongoing fat loss and fibrosis progression exhibited significantly elevated risks of all-cause mortality (HR: 2.14), liver-related events (HR: 1.77), and hepatic decompensation (HR: 1.83) [25]. Liver fat loss, particularly the ‘burnt-out’ phenotype, indicates disease progression and poor prognosis in advanced MASLD. Amelioration of steatosis, which often reflects cirrhosis, indicates worsening fibrosis rather than improvement. Thus, reductions in controlled attenuation parameter values or the amelioration of steatosis should not be misinterpreted as remission but rather considered a sign of advanced disease [25]. Accordingly, our patients having multiple CMRFs but no SLD might have previously developed steatosis. Through the steatotic ‘burnt-out’ mechanism, the absence of steatosis in patients with clustered cardiometabolic risk factors may reflect a subgroup with prior steatosis loss; however, this possibility remains speculative because longitudinal steatosis data were not available in the present study. Furthermore, although this concept has been described in non-viral MASLD cohorts, its applicability to antiviral-treated CHB remains uncertain and should be interpreted cautiously. Second, the prevalence of fibrosis in our cohort at baseline may be attributable to persistent HBV infection and associated chronic necroinflammatory activity. Hepatocyte fat loss occurred as fibrosis severity progressed during the course of CHB. A prospective study is required to validate our hypothesis.

Patients with CHB have varying risks of HCC, depending on the presence of SLD and CMRFs. The absence of SLD in patients with a high metabolic risk indicates an elevated risk of HCC, necessitating intensive surveillance. Conversely, the presence of SLD in patients with no clustered CMRFs indicates a reduced risk of HCC. These findings highlight the need for a tailored surveillance strategy for HCC. In addition to the burden of CHB, the rising burden of MASLD is driving a global increase in HCC incidence. Notably, HCC can develop even in noncirrhotic liver, complicating early detection and thus leading to late-stage diagnosis [26]. Previous models for HCC risk stratification in patients with CHB receiving antiviral therapy have primarily focused on clinical parameters and fibrosis burden (especially with baseline VCTE-determined LSM ≥11 kPa), lacking SLD and sufficient cardiometabolic information [27–30]. Recent multicenter studies have reinforced the prognostic relevance of metabolic disease burden in CHB [17,18]. The steatosis-associated fibrosis estimator (SAFE) score, initially proposed to distinguish significant fibrosis from stage 0 or 1 fibrosis in patients with nonalcoholic fatty liver disease, shows satisfactory performance for predicting HCC in patients with CHB as well as patients with MASLD, suggesting the potential role of steatosis and CMRFs in HBV-related HCC [31]. Our findings complement these reports by focusing specifically on the interaction between steatosis status and clustered cardiometabolic risk factors in NA-treated CHB. Future risk calculators should integrate SLD and metabolic burden to identify high-risk individuals for intensive surveillance and optimize resource allocation, aligning with the paradigm of precision medicine.

The present study has several strengths. It included a large, well-characterized cohort of NA-treated patients with CHB who had long-term follow-up data available. Rigorous statistical methods, including IPTW, minimized confounding effects and enabled robust subgroup analyses. Notably, the availability of longitudinal virological and fibrosis data allowed us to investigate the potential protective mechanisms of SLD.

This study has some limitations. First, because this was a single-center Taiwanese cohort from an Asian tertiary care center, the generalizability of the findings to other populations may be limited. Second, SLD was defined by ultrasonography rather than a quantitative modality; as ultrasonography may miss mild steatosis and cannot quantify fat burden, misclassification of low-grade or subclinical steatosis remains possible, particularly in the non-SLD group, and this methodological difference may partly explain discordance with CAP-based studies. Third, despite the use of IPTW, residual confounding from unmeasured lifestyle factors, medication exposures, and incompletely captured comorbidity management cannot be excluded. Fourth, temporal changes in steatosis status were not assessed because SLD was classified at baseline only; patients may have developed or lost steatosis during follow-up, introducing potential time-varying misclassification. Fifth, substantial missingness in fasting glucose, triglycerides, and HDL-C may have affected classification of cardiometabolic risk factor burden, and extensive imputation of these defining metabolic variables may introduce additional uncertainty in risk categorization. Sixth, because FIB-4 incorporates age and aminotransferase levels, between-group differences in these variables may have influenced fibrosis estimation; accordingly, FIB-4 and LSM were interpreted as noninvasive surrogate indices, and fibrosis-related inferences should be made cautiously in the absence of routine histologic confirmation. Seventh, systematic serologic assessment for occult autoimmune liver disease was unavailable, precluding evaluation of the potential impact of immunologic heterogeneity, including autoantibody positivity, on host response to HBV infection; this warrants further study [32]. Finally, the lack of mechanistic biomarker data precluded deeper investigation into the biological basis of the observed association between SLD and HCC risk.

## Conclusion

SLD reduced the risk of HCC in NA-treated patients with CHB, particularly those with advanced age, cirrhosis, and high metabolic risks. By contrast, the presence of ≥2 CMRFs, including DM, markedly increased the risk of HCC: patients without SLD having ≥2 CMRFs had the highest risk and most unfavorable clinical outcomes. These findings suggest both steatosis and metabolic burden should be incorporated into risk stratification models to refine surveillance strategies and advance CHB management in the direction of precision medicine. Further studies are required to unveil the mechanisms underlying the hepatoprotective effects of steatosis.

## Supplementary Material

Figure S1.tif

Supplementary Material for Review_R1.docx

Figure S2C.tif

Figure S2A.tif

Figure S2D.tif

Figure S2B.tif

## Data Availability

The authors confirm that the data supporting the findings of this study are available within the article and its Supporting Information. Data will be available upon reasonable request from the corresponding author of the manuscript.
