# Supplementary figures and images for: Absence of steatosis combined with cardiometabolic risk factors confers the highest hepatocellular carcinoma risk in treated chronic hepatitis B
Source: Ann Med. 2026 Apr 20;58(1):2658921. doi: 10.1080/07853890.2026.2658921 (PMC13097169; doi:10.1080/07853890.2026.2658921)

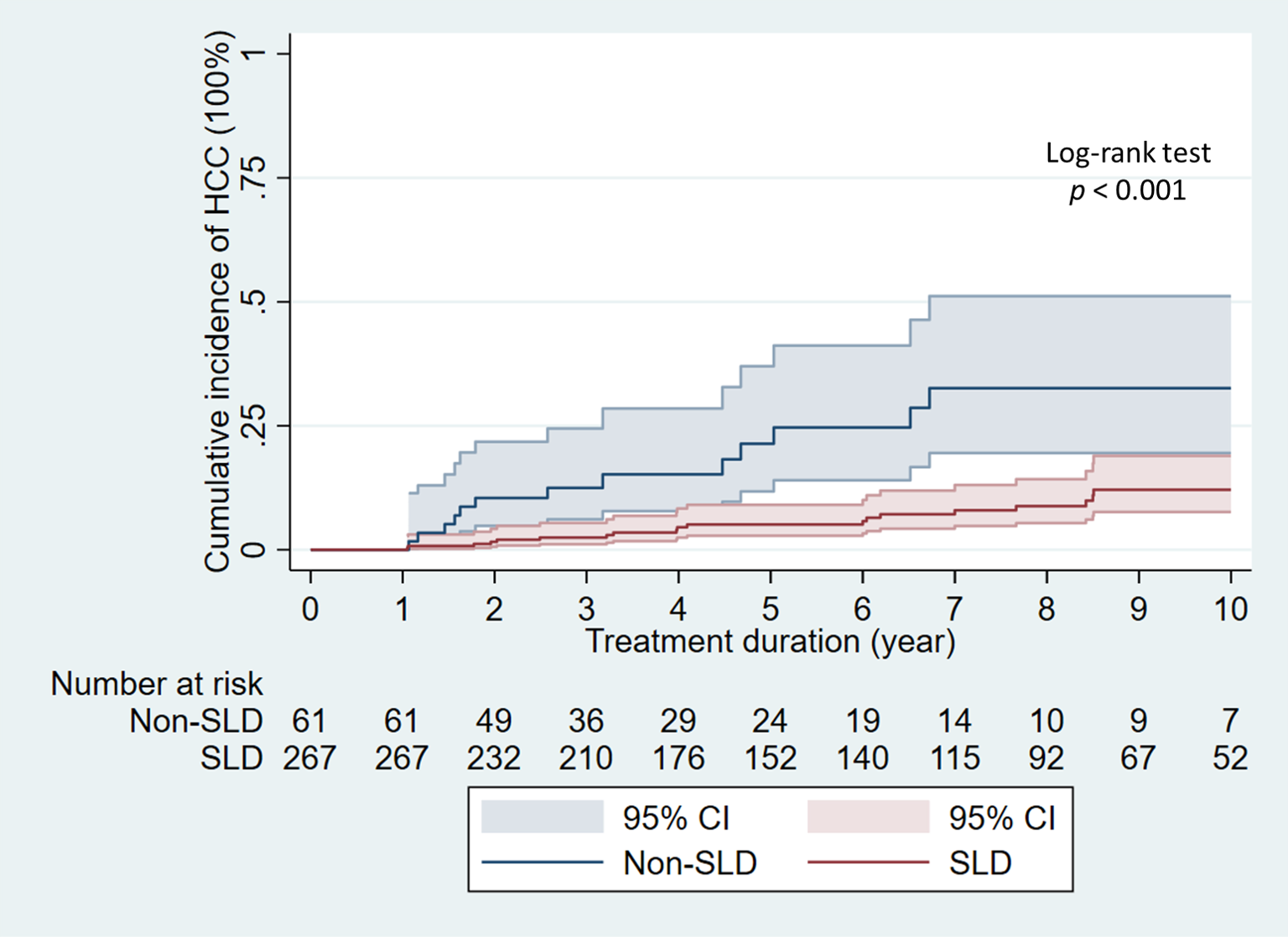

Supplement: Figure S1.tif [file IANN_A_2658921_SM4380.tif]

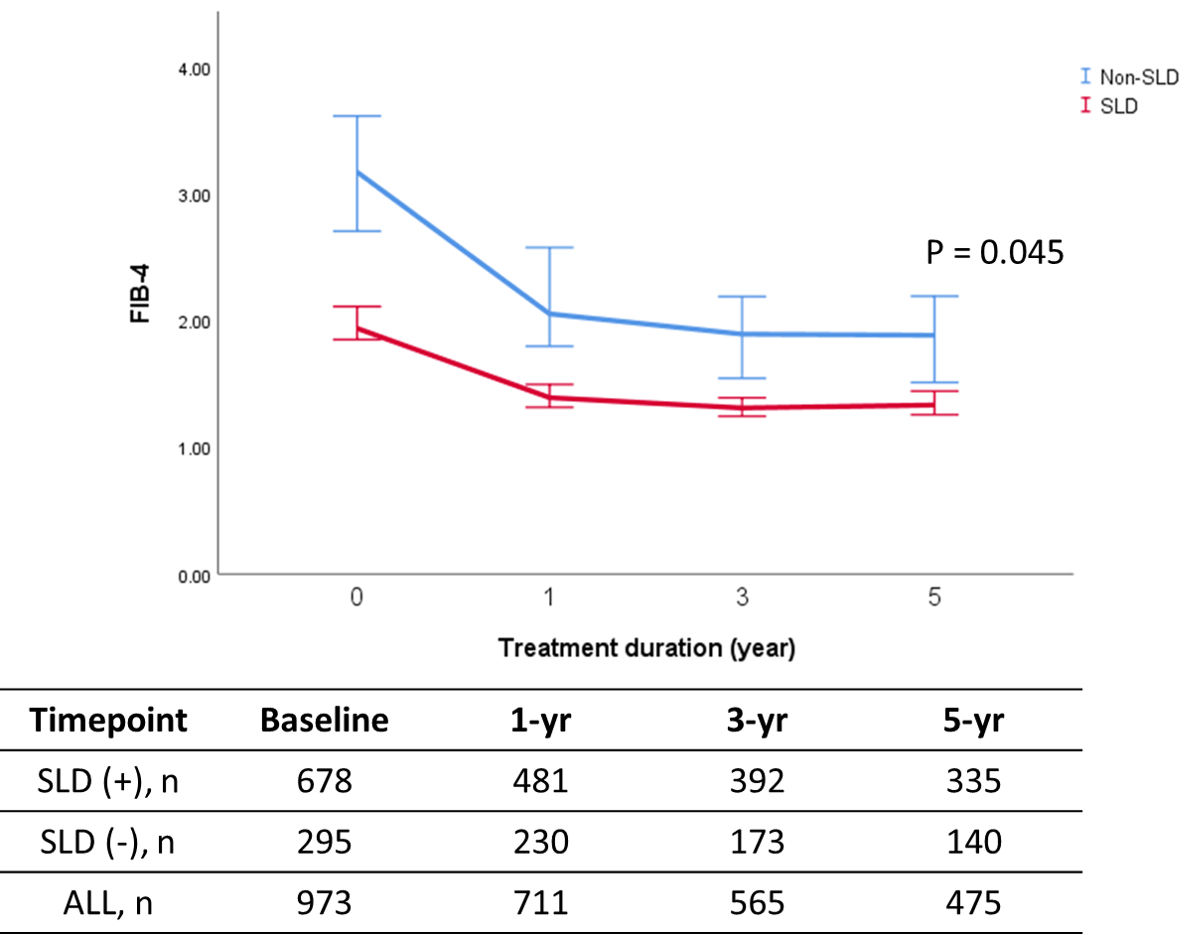

Supplement: Figure S2C.tif [file IANN_A_2658921_SM4378.tif]

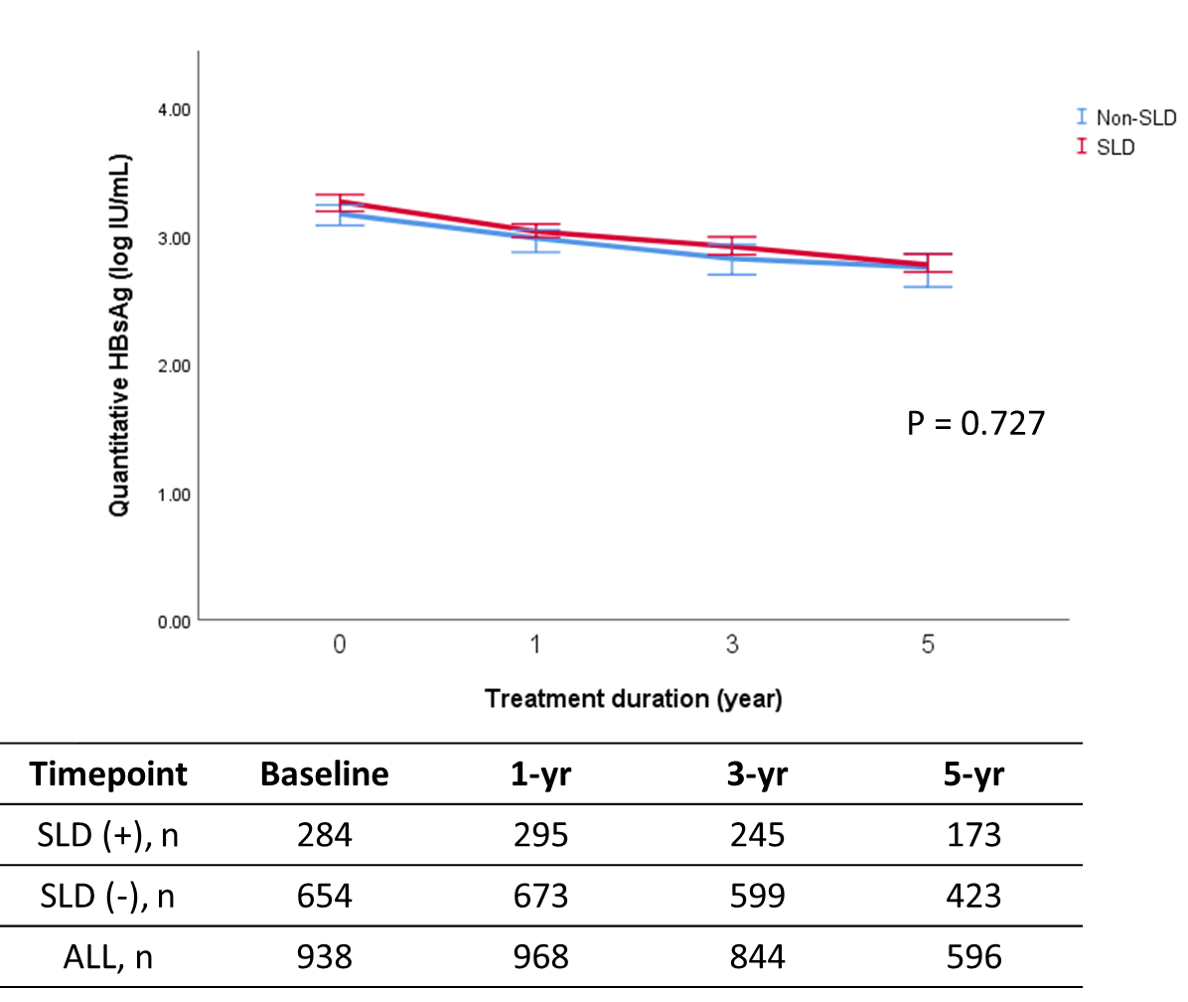

Supplement: Figure S2A.tif [file IANN_A_2658921_SM4377.tif]

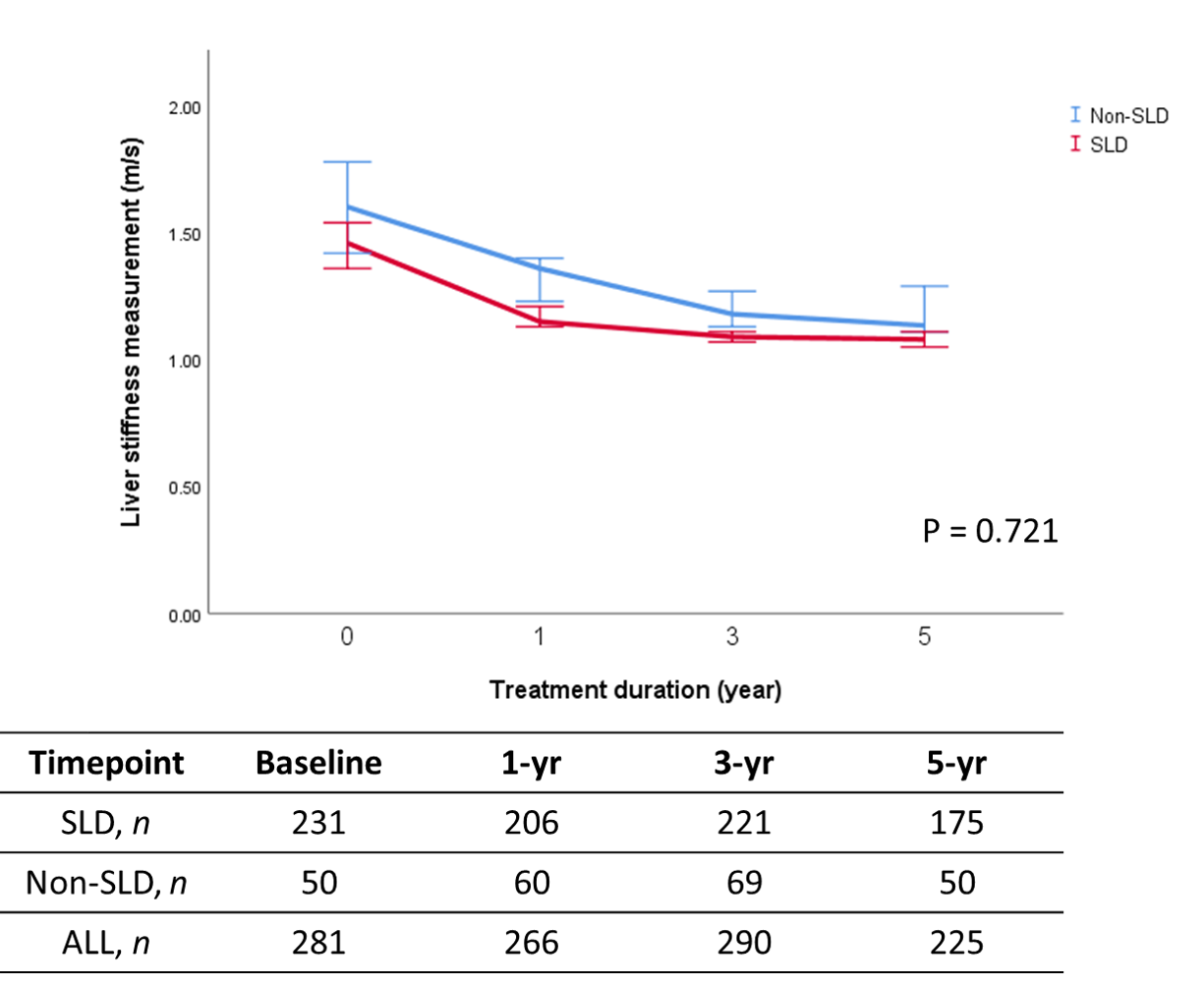

Supplement: Figure S2D.tif [file IANN_A_2658921_SM4376.tif]

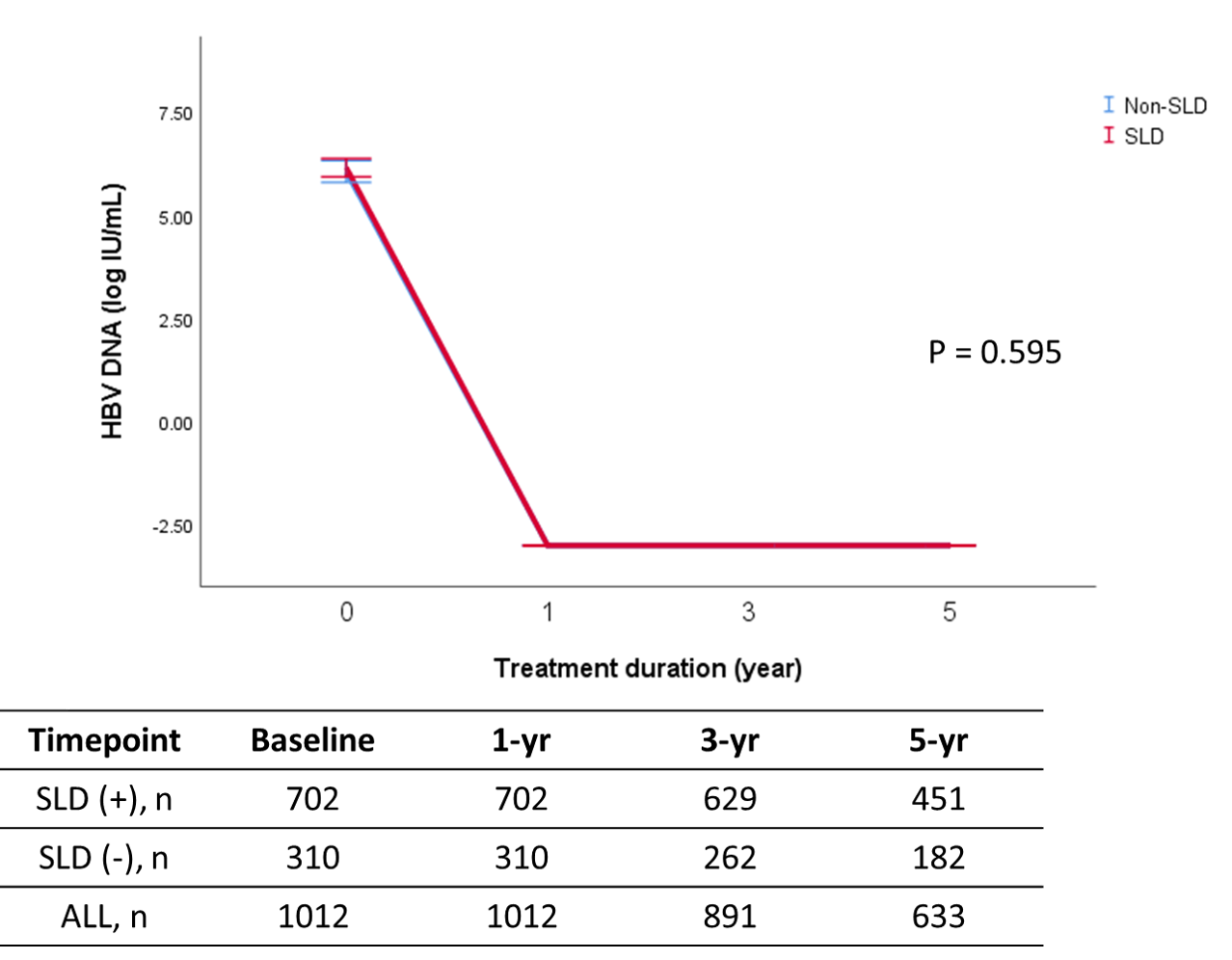

Supplement: Figure S2B.tif [file IANN_A_2658921_SM4375.tif]
