# Supplementary material for: Absence of steatosis combined with cardiometabolic risk factors confers the highest hepatocellular carcinoma risk in treated chronic hepatitis B
Source: Ann Med. 2026 Apr 20;58(1):2658921. doi: 10.1080/07853890.2026.2658921 (PMC13097169; doi:10.1080/07853890.2026.2658921)
Supplement: Supplementary Material for Review_R1.docx [file IANN_A_2658921_SM4379.docx]

**SUPPLEMENTARY TABLES**

**Supplementary Table 1.** Cardiometabolic risk factors associated with HCC incidence (*N* = 1012).

| **Variables** | **Crude Hazard Ratio (95% CI)** | ***p* value** | **Adjusted Hazard Ratio (95% CI)** | ***p* value** |
| --- | --- | --- | --- | --- |
| BMI ≥23 vs. <23 (kg/m^2^) | 1.567 (0.899–2.731) | 0.113 | 1.517 (0.869–2.646) | 0.142 |
| DM | 2.054 (1.217–3.465) | 0.007 | 1.903 (1.079–3.355) | 0.026 |
| Pre-DM and DM | 1.804 (1.136–2.864) | 0.012 |  |  |
| Hypertension | 1.375 (0.821–2.302) | 0.226 |  |  |
| TG^1^, mg/dL | 0.926 (0.289–2.969) | 0.897 |  |  |
| HDL-C^2^, mg/dL | 0.999 (0.352–2.833) | 0.998 |  |  |

^1^A plasma TG level of ≥150 mg/dL or the use of lipid-lowering drugs.

^2^A plasma HDL-C level of ≤40 mg/dL in men and ≤50 mg/dL in women or the use of lipid-lowering drugs.

Shaded cells indicate that the variable exerted a confounding effect on other factors and was therefore not included in the multivariate model.

Abbreviations: BMI, body mass index; CI, confidence interval; DM, diabetes mellitus; HDL-C, high-density lipoprotein cholesterol; HCC, hepatocellular carcinoma; TG, triglyceride.

**Supplementary Table 2.** Cardiometabolic risk factors associated with HCC incidence (*N* = 1012).

| **Variables** | **Crude Hazard Ratio (95% CI)** | ***p* value** | **Adjusted Hazard Ratio (95% CI)** | ***p* value** |
| --- | --- | --- | --- | --- |
| BMI ≥23 vs. <23 (kg/m^2^) | 1.567 (0.899–2.731) | 0.113 | 1.571 (0.902–2.738) | 0.111 |
| DM | 2.054 (1.217–3.465) | 0.007 |  |  |
| Pre-DM and DM | 1.804 (1.136–2.864) | 0.012 | 1.793 (1.094–2.938) | 0.021 |
| Hypertension | 1.375 (0.821–2.302) | 0.226 |  |  |
| TG^1^, mg/dL | 0.926 (0.289–2.969) | 0.897 |  |  |
| HDL-C^2^, mg/dL | 0.999 (0.352–2.833) | 0.998 |  |  |

^1^A plasma TG level of ≥150 mg/dL or the use of lipid-lowering drugs.

^2^A plasma HDL-C level of ≤40 mg/dL in men and ≤50 mg/dL in women or the use of lipid-lowering drugs.

Shaded cells indicate that the variable exerted a confounding effect on other factors and was therefore not included in the multivariate model.

Abbreviations: BMI, body mass index; CI, confidence interval; DM, diabetes mellitus; HDL-C, high-density lipoprotein cholesterol; HCC, hepatocellular carcinoma; TG, triglyceride.

**Supplementary Table 3.** Baseline clinicodemographic characteristics of patients with CHB having ≥2 CMRFs, stratified by SLD status (*N* = 328).

| **Parameter**  ***N* (%) or median [IQR]** | **CHB without SLD**  **(*N* = 61)** | **CHB with SLD**  **(*N* = 267)** | ***p* value** |
| --- | --- | --- | --- |
| Age, year | 60 [15.5] | 52 [15] | <0.001 |
| Male | 42 (68.9) | 199 (74.5) | 0.422 |
| BMI, kg/m^2^ | 24.5 [3.0] | 25.6 [3.9] | 0.020 |
| DM | 28 (45.9) | 97 (36.3) | 0.189 |
| Pre-DM and DM | 41 (67.2) | 187 (70.0) | 0.648 |
| Hypertension | 41 (67.2) | 133 (49.8) | 0.016 |
| Cirrhosis* | 35 (57.4) | 86 (32.2) | <0.001 |
| Alcohol | 13 (21.3) | 62 (23.2) | 0.866 |
| qHBsAg, log IU/mL | 2.90 [0.85] | 3.18 [0.98] | 0.084 |
| HBeAg, positivity | 10 (16.4) | 72 (26.9) | 0.101 |
| HBV DNA, log IU/mL | 5.50 [2.51] | 5.96 [2.33] | 0.448 |
| AST, U/L | 76.5 [183.3] | 65 [81.5] | 0.039 |
| ALT, U/L | 86 [326.5] | 96 [150] | 0.843 |
| Platelet count, 10^9^/L | 142 [88] | 169 [70] | 0.002 |
| AFP, ng/mL | 8.73 [19.4] | 5.29 [7.52] | 0.009 |
| FIB-4 | 3.90 [5.45] | 2.13 [2.37] | <0.001 |
| AC Glucose, mg/dL | 103 [22] | 104 [24] | 0.761 |
| TG^1^, mg/dL | 94 [62.5] | 105 [78.5] | 0.257 |
| HDL-C^2^, mg/dL | 50.5 [19.8] | 43.6 [16] | 0.034 |
| HCC incidence | 13 (21.3) | 20 (7.49) | 0.003 |

^1^A plasma TG level of ≥150 mg/dL or the use of lipid-lowering drugs.

^2^A plasma HDL-C level of ≤40 mg/dL in men or ≤50 mg/dL in women or the use of lipid-lowering drugs.

*Cirrhosis was diagnosed through ultrasonography or liver biopsy (*N* = 57).

Abbreviations: AC glucose, fasting (*ante cibum*) glucose; AFP, alpha-fetoprotein; ALT, alanine transaminase; AST, aspartate transaminase; BMI, body mass index; CHB, chronic hepatitis B; CMRF, cardiometabolic risk factor; DM, diabetes mellitus; FIB-4, fibrosis-4 index; HBeAg, hepatitis B e antigen; HBV, hepatitis B virus; HDL-C, high-density lipoprotein cholesterol; HCC, hepatocellular carcinoma; IQR, interquartile range; IU, international unit; PLT, platelet; Pre-DM, prediabetes; qHBsAg, quantitative hepatitis B surface antigen; SLD, steatotic liver disease; TG, triglyceride.

**Supplementary Table 4.** Factors associated with HCC incidence in patients with chronic hepatitis B having ≥2 CMRFs and receiving antiviral therapy.

| **Variables** | **Crude Hazard Ratio (95% CI)** | ***p* value** | **Adjusted Hazard Ratio (95% CI)** | ***p* value** |
| --- | --- | --- | --- | --- |
| Age ≥50 vs. <50 (year) | 1.849 (0.859–3.982) | 0.116 |  |  |
| Gender, female vs. male | 0.795 (0.345–1.833) | 0.591 |  |  |
| Alcohol | 1.168 (0.525–2.597) | 0.703 |  |  |
| SLD | 0.262 (0.130–0.529) | <0.001 | 0.262 (0.125–0.548) | <0.001 |
| BMI ≥23 vs. <23 (kg/m^2^) | 0.838 (0.321–2.186) | 0.718 |  |  |
| DM | 1.382 (0.692–2.758) | 0.360 |  |  |
| Pre-DM and DM | 1.297 (0.603–2.791) | 0.506 |  |  |
| Hypertension | 1.184 (0.588–2.383) | 0.637 |  |  |
| TG^1^, mg/dL | 0.878 (0.263–2.925) | 0.832 |  |  |
| HDL-C^2^, mg/dL | 0.816 (0.271–2.459) | 0.718 |  |  |
| HBeAg, positivity | 1.319 (0.639–2.724) | 0.454 |  |  |
| AST (per U/L increase) | 0.998 (0.996–1.001) | 0.165 |  |  |
| ALT (per U/L increase) | 0.998 (0.995–1.000) | 0.068 |  |  |
| PLT count (per 10^9^/L increase) | 0.984 (0.977–0.991) | <0.001 |  |  |
| AFP ≥9 vs. <9 (ng/mL) | 1.306 (0.650–2.626) | 0.454 |  |  |
| FIB-4 ≥3.25 vs. <3.25 | 1.833 (0.916–3.665) | 0.087 | 1.383 (0.673–2.842) | 0.377 |

^1^A plasma TG level of ≥150 mg/dL or the use of lipid-lowering drugs.

^2^A plasma HDL-C level of ≤40 mg/dL in men and ≤50 mg/dL in women or the use of lipid-lowering drugs.

Shaded cells indicate that the variable exerted a confounding effect on other factors and was therefore not included in the multivariate model.

Abbreviations: AFP, alpha-fetoprotein; ALT, alanine transaminase; AST, aspartate transaminase; BMI, body mass index; CI, confidence interval; CMRF, cardiometabolic risk factor; DM, diabetes mellitus; FIB-4, fibrosis-4 index; HDL-C, high-density lipoprotein cholesterol; HBeAg, hepatitis B e antigen; HCC, hepatocellular carcinoma; PLT, platelet; SLD, steatotic liver disease; TG, triglyceride.

**Supplementary Table 5.** Baseline clinicodemographic characteristics of patients with CHB having ≥2 CMRFs and receiving antiviral therapy, stratified by SLD status (before and after IPTW).

|  | **Before IPTW** |  |  | **After IPTW** |  |  |
| --- | --- | --- | --- | --- | --- | --- |
| **Parameter**  ***N* (%) or mean [SD]** | **CHB without SLD**  **(*N* = 61)** | **CHB with SLD**  **(*N* = 267)** | ***p* value** | **CHB without SLD**  **(*N* = 252)** | **CHB with SLD**  **(*N* = 300)** | ***p* value** |
| Age, year | 58.5 [11.8] | 52.4 [9.9] | <0.001 | 54.1 [11.9] | 53.6 [10.2] | 0.613 |
| Male | 42 (68.9) | 199 (74.5) | 0.422 | 182 (72.2) | 219 (73.0) | 0.849 |
| Alcohol | 13 (21.3) | 62 (23.2) | 0.866 | 57 (22.6) | 67 (22.3) | >0.999 |
| BMI, kg/m^2^ | 25.2 [3.4] | 25.9 [3.1] | 0.090 | 24.9 [2.7] | 25.9 [3.1] | <0.001 |
| DM | 28 (45.9) | 97 (36.3) | 0.189 | 107 (42.5) | 109 (36.5) | 0.162 |
| Pre-DM and DM | 41 (67.2) | 187 (70) | 0.648 | 173 (68.7) | 204 (68.0) | 0.927 |
| Hypertension | 41 (67.2) | 133 (49.8) | 0.016 | 165 (65.2) | 166 (55.3) | 0.019 |
| Cirrhosis* | 35 (57.4) | 86 (32.2) | <0.001 | 96 (37.9) | 117 (39.0) | 0.861 |
| qHBsAg, log IU/mL | 2.92 [0.84] | 3.08 [0.91] | 0.224 | 3.10 [0.79] | 3.05 [0.94] | 0.499 |
| HBeAg, positivity | 10 (16.4) | 72 (27.0) | 0.101 | 60 (23.8) | 80 (26.7) | 0.492 |
| HBV DNA, log IU/mL | 5.84 [1.43] | 5.99 [1.45] | 0.471 | 6.07 [1.49] | 5.96 [1.45] | 0.405 |
| AST, U/L | 216.1 [309.9] | 147.2 [249.5] | 0.066 | 216.6 [287.3] | 184.3 [315.8] | 0.213 |
| ALT, U/L | 253.8 [326.5] | 206.6 [298.2] | 0.274 | 291.1 [328.1] | 231.7 [326.9] | 0.034 |
| Platelet count, 10^9^/L | 144.6 [54.5] | 171.3 [56.4] | <0.001 | 158.3 [53.9] | 165.5 [55.9] | 0.129 |
| AFP, ng/mL | 30.6 [69.7] | 39.5 [190.7] | 0.725 | 29.0 [67.1] | 39.7 [180.0] | 0.373 |
| FIB-4 | 6.34 [7.12] | 3.32 [3.66] | <0.001 | 4.59 [4.81] | 4.24 [5.60] | 0.438 |
| AC Glucose, mg/dL | 122.5 [48.1] | 116.1 [44.5] | 0.418 | 112.3 [35.9] | 116.2 [46.5] | 0.350 |
| TG^1^, mg/dL | 112.7 [76.6] | 121.9 [76.5] | 0.462 | 107.4 [77.0] | 122.2 [76.7] | 0.050 |
| HDL-C^2^, mg/dL | 51.9 [17.2] | 46.2 [12.9] | 0.032 | 50.5 [13.5] | 46.4 [13.3] | 0.005 |
| HCC incidence | 13 (21.3) | 20 (7.5) | 0.003 | 47 (18.6) | 24 (8.0) | <0.001 |

^1^A plasma TG level of ≥150 mg/dL or the use of lipid-lowering drugs.

^2^A plasma HDL-C level of ≤40 mg/dL in men or ≤50 mg/dL in women or the use of lipid-lowering drugs.

*Cirrhosis was diagnosed through ultrasonography or liver biopsy (n = 57).

Abbreviations: AC glucose, fasting (*ante cibum*) glucose; AFP, alpha-fetoprotein; ALT, alanine transaminase; AST, aspartate transaminase; BMI, body mass index; CHB, chronic hepatitis B; DM, diabetes mellitus; FIB-4, fibrosis-4 index; HBeAg, hepatitis B e antigen; HBV, hepatitis B virus; HDL-C, high-density lipoprotein cholesterol; HCC, hepatocellular carcinoma; IPTW, inverse probability of treatment weighting; IU, international unit; PLT, platelet; Pre-DM, prediabetes; qHBsAg, quantitative hepatitis B surface antigen; SD, standard deviation; SLD, steatotic liver disease; TG, triglyceride.

**Supplementary Table 6.** Factors associated with HCC incidence after IPTW in patients with chronic hepatitis B having ≥2 CMRFs and receiving antiviral therapy.

| **Variables** | **Crude Hazard Ratio (95% CI)** | ***p* value** | **Adjusted Hazard Ratio (95% CI)** | ***p* value** |
| --- | --- | --- | --- | --- |
| Age ≥50 vs. <50 (year) | 1.788 (0.603–5.304) | 0.295 |  |  |
| Gender, female vs. male | 0.779 (0.262–2.317) | 0.653 |  |  |
| Alcohol | 0.848 (0.295–2.440) | 0.760 |  |  |
| SLD | 0.382 (0.172–0.852) | 0.019 | 0.382 (0.172–0.852) | 0.019 |
| BMI ≥23 vs. <23 (kg/m^2^) | 0.839 (0.220–3.200) | 0.797 |  |  |
| DM | 1.431 (0.593–3.450) | 0.425 |  |  |
| Pre-DM and DM | 1.106 (0.424–2.881) | 0.837 |  |  |
| Hypertension | 1.063 (0.437–2.584) | 0.894 |  |  |
| TG^1^, mg/dL | 0.937 (0.238–3.693) | 0.926 |  |  |
| HDL-C^2^, mg/dL | 1.863 (0.538–6.449) | 0.326 |  |  |
| HBeAg, positivity | 0.802 (0.328–1.961) | 0.629 |  |  |
| AST (per U/L increase) | 0.997 (0.994–1.000) | 0.051 |  |  |
| ALT (per U/L increase) | 0.997 (0.994–0.999) | 0.037 |  |  |
| PLT count (per 10^9^/L increase) | 0.987 (0.977–0.997) | 0.011 |  |  |
| AFP ≥9 vs. <9 (ng/mL) | 1.035 (0.424–2.524) | 0.941 |  |  |
| FIB-4 ≥3.25 vs. <3.25 | 1.630 (0.661–4.022) | 0.289 |  |  |

^1^A plasma TG level of ≥150 mg/dL or the use of lipid-lowering drugs.

^2^A plasma HDL-C level of ≤40 mg/dL in men and ≤50 mg/dL in women or the use of lipid-lowering drugs.

Shaded cells indicate that the variable exerted a confounding effect on other factors and was therefore not included in the multivariate model.

Abbreviations: AFP, alpha-fetoprotein; ALT, alanine transaminase; AST, aspartate transaminase; BMI, body mass index; CI, confidence interval; CMRF, cardiometabolic risk factor; DM, diabetes mellitus; FIB-4, fibrosis-4 index; HDL-C, high-density lipoprotein cholesterol; HBeAg, hepatitis B e antigen; HCC, hepatocellular carcinoma; IPTW, inverse probability of treatment weighting; PLT, platelet; SLD, steatotic liver disease; TG, triglyceride.

**Supplementary Table 7.** Longitudinal levels of FIB-4 and LSM in patients with CHB with or without SLD.

| **Parameter** | **Sample number** | **CHB without SLD** | **CHB with SLD** | ***p* value** |
| --- | --- | --- | --- | --- |
| **FIB-4** | ***N*** | **median [IQR]** | **median [IQR]** |  |
| ***ALL patients*** | 1012 |  |  |  |
| at baseline | 973 | 3.17 [4.90] | 1.94 [2.01] | <0.001 |
| at 1-year | 711 | 2.05 [2.91] | 1.39 [1.16] | <0.001 |
| at 3-year | 565 | 1.89 [2.37] | 1.31 [1.00] | <0.001 |
| at 5-year | 475 | 1.88 [1.95] | 1.33 [0.95] | <0.001 |
| ***Subgroup: age*** *≥****50*** | 480 |  |  |  |
| at baseline | 466 | 4.42 [5.46] | 2.45 [2.16] | <0.001 |
| at 1-year | 369 | 3.05 [3.08] | 1.81 [1.22] | <0.001 |
| at 3-year | 285 | 2.42 [2.36] | 1.71 [1.06] | <0.001 |
| at 5-year | 224 | 2.50 [2.29] | 1.80 [1.26] | <0.001 |
| ***Subgroup: cirrhosis*** | 312 |  |  |  |
| at baseline | 303 | 5.31 [5.80] | 2.38 [2.57] | <0.001 |
| at 1-year | 258 | 3.71 [3.66] | 2.08 [2.06] | <0.001 |
| at 3-year | 210 | 3.22 [3.50] | 1.89 [1.52] | <0.001 |
| at 5-year | 200 | 2.84 [3.48] | 1.68 [1.44] | <0.001 |
| ***Subgroup: CMRF*** *≥****1*** | 759 |  |  |  |
| at baseline | 736 | 3.89 [5.45] | 1.95 [1.97] | <0.001 |
| at 1-year | 541 | 2.73 [3.16] | 1.41 [1.16] | <0.001 |
| at 3-year | 433 | 2.21 [2.41] | 1.30 [1.05] | <0.001 |
| at 5-year | 379 | 2.24 [2.24] | 1.33 [0.95] | <0.001 |
| ***Subgroup: CMRF*** *≥****2*** | 328 |  |  |  |
| at baseline | 316 | 3.90 [5.45] | 2.13 [2.44] | <0.001 |
| at 1-year | 234 | 2.90 [2.41] | 1.59 [1.23] | <0.001 |
| at 3-year | 187 | 2.24 [2.12] | 1.42 [1.01] | <0.001 |
| at 5-year | 171 | 2.19 [3.22] | 1.43 [0.89] | <0.001 |
| **LSM** | ***N*** | **median [IQR]** | **median [IQR]** |  |
| ***ALL patients*** | 1012 |  |  |  |
| at baseline | 281 | 1.60 [0.71] | 1.46 [0.92] | 0.210 |
| at 1-year | 266 | 1.36 [0.42] | 1.15 [0.31] | <0.001 |
| at 3-year | 290 | 1.18 [0.43] | 1.09 [0.23] | 0.006 |
| at 5-year | 225 | 1.14 [0.26] | 1.08 [0.25] | 0.004 |
| ***Subgroup: age*** *≥****50*** | 480 |  |  |  |
| at baseline | 130 | 1.55 [0.86] | 1.52 [0.91] | 0.511 |
| at 1-year | 140 | 1.38 [0.87] | 1.20 [0.34] | 0.002 |
| at 3-year | 139 | 1.22 [0.49] | 1.11 [0.27] | 0.043 |
| at 5-year | 104 | 1.14 [0.34] | 1.07 [0.25] | 0.026 |
| ***Subgroup: cirrhosis*** | 312 |  |  |  |
| at baseline | 102 | 1.87 [0.92] | 1.96 [1.05] | 0.882 |
| at 1-year | 85 | 1.51 [0.75] | 1.37 [0.87] | 0.131 |
| at 3-year | 95 | 1.42 [0.46] | 1.27 [0.43] | 0.363 |
| at 5-year | 87 | 1.33 [0.72] | 1.22 [0.33] | 0.017 |
| ***Subgroup: CMRF*** *≥****1*** | 759 |  |  |  |
| at baseline | 225 | 1.78 [1.09] | 1.48 [1.05] | 0.154 |
| at 1-year | 204 | 1.39 [0.94] | 1.14 [0.36] | 0.001 |
| at 3-year | 229 | 1.22 [0.59] | 1.09 [0.23] | 0.030 |
| at 5-year | 184 | 1.14 [0.55] | 1.07 [0.25] | 0.027 |
| ***Subgroup: CMRF*** *≥****2*** | 328 |  |  |  |
| at baseline | 112 | 2.24 [1.12] | 1.83 [1.34] | 0.358 |
| at 1-year | 101 | 1.40 [1.44] | 1.16 [0.56] | 0.009 |
| at 3-year | 106 | 1.41 [0.56] | 1.10 [0.42] | 0.065 |
| at 5-year | 82 | 1.30 [1.08] | 1.07 [0.34] | 0.023 |

Abbreviations: CHB, chronic hepatitis B; FIB-4, fibrosis-4 index; HBV, hepatitis B virus; IQR, interquartile range; IU, international unit; qHBsAg, quantitative hepatitis B surface antigen; SLD, steatotic liver disease.

**Supplementary Table 8.** Longitudinal levels of qHBsAg and HBV DNA in patients with CHB with or without SLD.

| **Parameter** | **Sample number** | **CHB without SLD** | **CHB with SLD** | ***p* value** |
| --- | --- | --- | --- | --- |
| **qHBsAg, log IU/mL** | ***N*** | **median [IQR]** | **median [IQR]** |  |
| ***ALL patients*** | 1012 |  |  |  |
| at baseline | 938 | 3.17 [0.87] | 3.27 [0.93] | 0.064 |
| at 1-year | 968 | 2.98 [0.73] | 3.03 [0.74] | 0.009 |
| at 3-year | 844 | 2.82 [0.82] | 2.91 [0.76] | 0.021 |
| at 5-year | 596 | 2.75 [1.06] | 2.77 [0.83] | 0.306 |
| ***Subgroup: age*** *≥****50*** | 480 |  |  |  |
| at baseline | 446 | 2.89 [0.68] | 3.06 [0.87] | 0.012 |
| at 1-year | 462 | 2.78 [0.68] | 2.85 [0.77] | 0.107 |
| at 3-year | 389 | 2.58 [0.82] | 2.67 [0.75] | 0.083 |
| at 5-year | 275 | 2.40 [0.88] | 2.55 [0.74] | 0.023 |
| ***Subgroup: cirrhosis*** | 312 |  |  |  |
| at baseline | 292 | 3.00 [0.81] | 3.18 [0.63] | 0.005 |
| at 1-year | 299 | 2.82 [0.82] | 3.05 [0.58] | <0.001 |
| at 3-year | 264 | 2.65 [0.95] | 2.90 [0.64] | 0.001 |
| at 5-year | 217 | 2.47 [1.03] | 2.71 [0.75] | 0.026 |
| ***Subgroup: CMRF*** *≥****1*** | 759 |  |  |  |
| at baseline | 710 | 3.10 [0.97] | 3.23 [0.93] | 0.012 |
| at 1-year | 727 | 2.92 [0.76] | 3.01 [0.73] | 0.008 |
| at 3-year | 640 | 2.72 [0.90] | 2.87 [0.75] | 0.005 |
| at 5-year | 461 | 2.60 [1.14] | 2.73 [0.81] | 0.026 |
| ***Subgroup: CMRF*** *≥****2*** | 328 |  |  |  |
| at baseline | 312 | 2.90 [0.85] | 3.18 [0.98] | 0.084 |
| at 1-year | 315 | 2.78 [0.77] | 2.91 [0.80] | 0.029 |
| at 3-year | 281 | 2.52 [0.85] | 2.76 [0.73] | 0.005 |
| at 5-year | 210 | 2.06 [1.03] | 2.61 [0.79] | 0.002 |
| **HBV DNA, log IU/mL** | ***N*** | **median [IQR]** | **median [IQR]** |  |
| ***ALL patients*** | 1012 |  |  |  |
| at baseline | 1012 | 6.05 [2.41] | 6.16 [2.51] | 0.572 |
| at 1-year | 1012 | -3 [0] | -3 [0] | 0.254 |
| at 3-year | 891 | -3 [0] | -3 [0] | 0.044 |
| at 5-year | 633 | -3 [0] | -3 [0] | 0.317 |
| ***Subgroup: age*** *≥****50*** | 480 |  |  |  |
| at baseline | 480 | 5.54 [1.94] | 5.80 [2.03] | 0.195 |
| at 1-year | 480 | -3 [0] | -3 [0] | 0.340 |
| at 3-year | 419 | -3 [0] | -3 [0] | 0.193 |
| at 5-year | 295 | -3 [0] | -3 [0] | 0.220 |
| ***Subgroup: cirrhosis*** | 312 |  |  |  |
| at baseline | 312 | 5.35 [2.16] | 5.62 [2.04] | 0.360 |
| at 1-year | 312 | -3 [0] | -3 [0] | 0.135 |
| at 3-year | 275 | -3 [0] | -3 [0] | 0.898 |
| at 5-year | 231 | -3 [0] | -3 [0] | 0.465 |
| ***Subgroup: CMRF*** *≥****1*** | 759 |  |  |  |
| at baseline | 759 | 5.91 [2.34] | 6.11 [2.49] | 0.178 |
| at 1-year | 759 | -3 [0] | -3 [0] | 0.058 |
| at 3-year | 672 | -3 [0] | -3 [0] | 0.051 |
| at 5-year | 492 | -3 [0] | -3 [0] | 0.333 |
| ***Subgroup: CMRF*** *≥****2*** | 328 |  |  |  |
| at baseline | 328 | 5.50 [2.51] | 5.96 [2.33] | 0.448 |
| at 1-year | 328 | -3 [0] | -3 [0] | 0.061 |
| at 3-year | 294 | -3 [0] | -3 [0] | 0.161 |
| at 5-year | 220 | -3 [0] | -3 [0] | 0.298 |

Abbreviations: CHB, chronic hepatitis B; FIB-4, fibrosis-4 index; HBV, hepatitis B virus; IQR, interquartile range; IU, international unit; LSM, liver stiffness measurement; qHBsAg, quantitative hepatitis B surface antigen; SLD, steatotic liver disease.

**Supplementary Table 9.** Dynamic changes (percentage) in qHBsAg, HBV DNA, and FIB-4 in patients with CHB with or without SLD.

| **Parameter** | **Sample number** | **CHB without SLD** | **CHB with SLD** | ***p* value** |
| --- | --- | --- | --- | --- |
| **Δ FIB-4 (%)** | ***N*** | **median [IQR]** | **median [IQR]** |  |
| at 1-year (0-1yr) | 685 | -26.0 [50.3] | -24.5 [43.6] | 0.981 |
| at 3-year (0-3yr) | 549 | -32.9 [58.2] | -28.8 [44.7] | 0.107 |
| at 5-year (0-5yr) | 464 | -32.1 [59.2] | -29.6 [44.2] | 0.174 |
| **Δ LSM (%)** | ***N*** | **median [IQR]** | **median [IQR]** |  |
| at 1-year (0-1yr) | 204 | -11.4 [22.8] | -14.5 [26.1] | 0.764 |
| at 3-year (0-3yr) | 211 | -19.6 [20.3] | -22.9 [28.7] | 0.301 |
| at 5-year (0-5yr) | 156 | -19.6 [17.6] | -27.1 [34.2] | 0.137 |
| **Δ qHBsAg (%)** | ***N*** | **median [IQR]** | **median [IQR]** |  |
| at 1-year (0-1yr) | 912 | -26.3 [85.6] | -22.6 [79.6] | 0.202 |
| at 3-year (0-3yr) | 796 | -52.6 [72.9] | -44.9 [72.0] | 0.161 |
| at 5-year (0-5yr) | 564 | -61.5 [67.3] | -64.0 [56.1] | 0.682 |
| **Δ HBV DNA (%)** | ***N*** | **median [IQR]** | **median [IQR]** |  |
| at 1-year (0-1yr) | 1012 | -100 [0] | -100 [0] | 0.339 |
| at 3-year (0-3yr) | 891 | -100 [0] | -100 [0] | 0.583 |
| at 5-year (0-5yr) | 633 | -100 [0] | -100 [0] | 0.879 |

Abbreviations: CHB, chronic hepatitis B; FIB-4, fibrosis-4 index; HBV, hepatitis B virus; IQR, interquartile range; IU, international unit; LSM, liver stiffness measurement; qHBsAg, quantitative hepatitis B surface antigen; SLD, steatotic liver disease.

**SUPPLEMENTARY FIGRUE LEGEND**

**Supplementary Figure S1.** Cumulative incidence of HCC by SLD status in CHB patients with multiple CMRFs (log-rank *p*<0.001). Shaded areas indicate 95% CIs; numbers at risk are shown. Abbreviations: HCC, hepatocellular carcinoma; SLD, steatotic liver disease; CHB, chronic hepatitis B; CMRFs, cardiometabolic risk factors; CI, confidence interval.

**Supplementary Figure** **S2.** Longitudinal changes in virological markers and fibrosis indices by SLD status during nucleos(t)ide analogue therapy. (A) Quantitative hepatitis B surface antigen (HBsAg; log IU/mL) over treatment duration in patients with SLD versus non-SLD. (B) Serum HBV DNA (log IU/mL) over treatment duration in patients with SLD versus non-SLD. (C) FIB-4 index over treatment duration in patients with SLD versus non-SLD. (D) Liver stiffness measurement by transient elastography (m/s) over treatment duration in patients with SLD versus non-SLD. Abbreviations: SLD, steatotic liver disease; HBsAg, hepatitis B surface antigen; HBV, hepatitis B virus; FIB-4, Fibrosis-4 index.
